# Cardiorespiratory fitness and pain severity: Longitudinal associations from the general population—The HUNT study

**DOI:** 10.1097/PR9.0000000000001447

**Published:** 2026-04-28

**Authors:** Live Førland Havstad, Astrid Woodhouse, Tormod Landmark, Dorthe Stensvold, Bjarne Nes, Martin Skagseth, Mari Glette

**Affiliations:** a Department of Circulation and Medical Imaging, Norwegian University of Science and Technology, Trondheim, Norway; b National Competence Centre for Complex Symptom Disorders, St. Olav's University Hospital, Trondheim, Norway; c NORCE Norwegian Research Centre, Bergen, Norway

**Keywords:** Chronic pain, Pain severity, Cardiorespiratory fitness, General population

## Abstract

**Introduction:**

Reduced cardiorespiratory fitness (CRF) is associated with chronic pain, but longitudinal population-based evidence is limited. Whether CRF can prevent pain worsening or promote improvement is unclear.

**Objective:**

This study investigated (1) the cross-sectional association between estimated CRF (eCRF) and chronic pain, (2) the longitudinal association between eCRF and changes in pain severity over 11 years, and (3) whether changes in eCRF are associated with changes in pain severity.

**Method:**

Data were collected from the Trøndelag Health Study (HUNT) in Norway. Cross-sectional analyses included 18,837 adults from HUNT3 (2006–08), and longitudinal analyses followed participants to HUNT4 (2017–19), stratified by baseline pain status: no or mild (n = 11,517) or moderate/severe chronic pain (n = 5312). Cardiorespiratory fitness was estimated using a validated nonexercise algorithm and categorized into sex- and age-specific quintiles. Logistic regression estimated odds ratios with 95% confidence intervals.

**Results:**

Cross-sectionally, higher eCRF was associated with lower odds of chronic pain, with the largest difference between the highest and the lowest quintiles (women: OR 0.56, 95% CI 0.49–0.64; men: OR 0.64, 95% CI 0.53–0.78). Longitudinally, among participants with no or mild pain, both high baseline eCRF and maintained high eCRF were associated with lower odds of pain worsening. Among those with chronic pain, maintaining or increasing eCRF was associated with greater odds of pain reduction, whereas high baseline eCRF showed a nonsignificant trend in the same direction.

**Conclusion:**

Higher eCRF was associated with lower odds of chronic pain and with more favorable changes in pain severity in the general population.

## Abstract 2

Supplemental Digital Content is Available in the Text.

Higher estimated cardiorespiratory fitness is associated with lower odds of chronic pain and with more favorable changes in pain severity in the general population.

## 1. Introduction

Chronic pain is a major public health challenge, with chronic primary pain now officially classified as a distinct diagnosis.^8,53^ According to the Global Burden of Diseases (GBD), it is among the leading causes of years lived with disability worldwide.^16^ The International Association for the Study of Pain (IASP) defines chronic pain as persistent or recurrent pain lasting more than 3 months,^54^ and recommends a 6-month cutoff for research purposes.^1^ Moderate to severe chronic pain affects approximately 30% of adults (5, 6), with musculoskeletal pain accounting for most cases (7). While most chronic pain conditions are not life threatening,^16^ they substantially impair quality of life,^19,36^ increase healthcare utilization,^12^ and contribute to work absence.^52^ Given these substantial individual and societal costs, there is an urgent need to identify modifiable risk factors and better understand how they influence the severity of pain over time.

Cardiorespiratory fitness (CRF) is a strong predictor of health and survival, with higher CRF associated with reduced all-cause mortality, independent of traditional risk factors.^39^ Reduced CRF has been observed in various chronic pain conditions, including low back pain,^48^ ankylosing spondylitis,^21^ fibromyalgia,^15^ and headache disorders.^20^ However, most studies are diagnosis-specific,^20–22,48–50,55,61^ and evidence from general population samples remains limited. The gold standard for measuring CRF is maximal oxygen uptake (VO_2max_) during a maximal cardiopulmonary exercise test. However, due to the cost, time, and need for trained personnel, various nonexercise models to calculate CRF have emerged.^42,43,56^ These models make it easier to investigate CRF through estimated CRF (eCRF) in large population samples. Physical activity has been more extensively studied than CRF in population samples. Higher activity levels are generally associated with lower chronic pain prevalence^14,17,23,32^ and greater pain tolerance.^64^ However, the evidence is ambiguous: while 1 study reported a dose–response relationship,^14^ another found a U-shaped association, with very high activity levels associated with increased prevalence of chronic pain.^32^

This study investigates the association between eCRF and pain severity in the general population. First, we investigate the cross-sectional association between eCRF and moderate to severe chronic pain. Second, we investigate the longitudinal association between eCRF and changes in pain severity over 11 years. Specifically, we examine how baseline levels of eCRF relate to an increase in pain severity among those with no or mild pain and a decrease in those with moderate to severe chronic pain at baseline. Third, we investigate how changes in eCRF relate to changes in pain severity separately for each of these 2 groups.

## 2. Method

### 2.1. Study design and participants

The HUNT study is a population-based cohort from former Nord-Trøndelag County, Norway, comprising 4 surveys: HUNT1 (1984–86), HUNT2 (1995–97), HUNT3 (2006–08), and HUNT4 (2017–19). All residents aged ≥20 years were invited, and more than 229,000 individuals have participated to date.^65^ The region is broadly representative of Norway.^65^ This study uses HUNT3 as baseline (n = 50,800; 54.1% participation) and HUNT4 as 11-year follow-up (n = 56,042; 54% participation). Data were collected through questionnaires, interviews, biological samples, and clinical examinations; further details are reported elsewhere.^29,65^

#### 2.1.1. Participants in the cross-sectional analysis

The cross-sectional analysis included participants who attended both HUNT3 and HUNT4 (n = 33,907). We excluded those with missing chronic pain status/pain severity (n = 2762), missing eCRF algorithm variables (n = 6961), or missing covariates (n = 5347), yielding a final sample of 18,837 (11,077 women; 7760 men).

#### 2.1.2. Participants in the longitudinal analysis

Longitudinal analyses also included HUNT3–HUNT4 participants (n = 33,907), dichotomized at baseline into (1) no or mild pain and (2) moderate to severe chronic pain.

##### 2.1.2.1. No or mild pain group

After excluding those with moderate to severe pain (n = 12,741) and missing baseline pain severity (n = 2426), we further excluded participants with missing follow-up pain severity (n = 341), eCRF variables (n = 3779), or covariates (n = 3103), leaving 11,517 (6463 women; 5054 men). For change in eCRF analyses, additional exclusions for missing HUNT4 eCRF variables (n = 1870) yielded 9647 (5383 women; 4264 men).

##### 2.1.2.2. Moderate to severe chronic pain group

After excluding those without moderate to severe chronic pain (n = 21,647) and those with missing chronic pain duration/severity (n = 2762), we excluded participants with missing follow-up pain severity (n = 244), eCRF variables (n = 2303), or covariates (n = 1639), leaving 5312 (3458 women; 1854 men). For change in eCRF analyses, additional exclusions for missing HUNT4 eCRF variables (n = 1074) yielded 4238 (2708 women; 1530 men).

**Figure 1.**
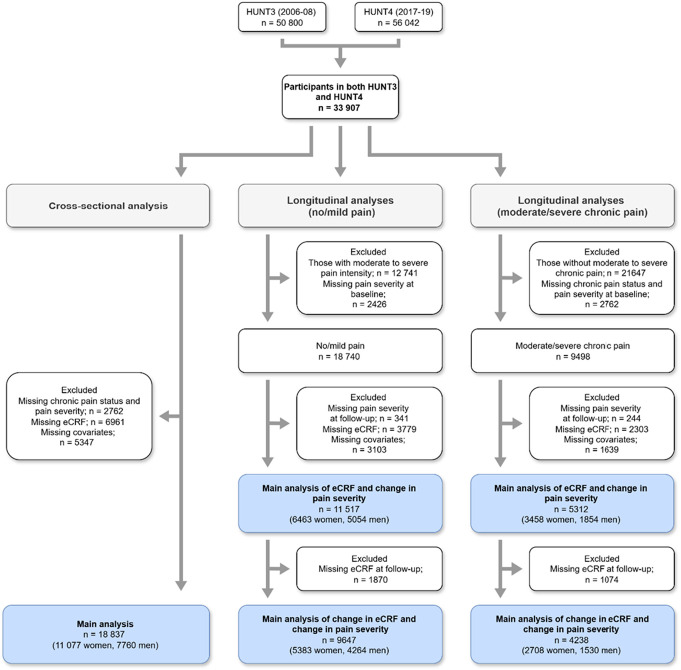
Flowchart of participant selection for cross-sectional and longitudinal analyses.

A flowchart of the selection process is presented in Figure 1.

### 2.2. Measures

#### 2.2.1. Classification of chronic pain and pain severity

Chronic pain duration was assessed by the question: “Do you have bodily pain that has lasted for more than 6 months?” derived from the Danish Health Survey^11^ and consistent with IASP recommendations for research purposes.^1^ Pain severity in HUNT3 and HUNT4 was assessed by the following: “How much bodily pain have you had during the past 4 weeks?” with responses on the Bodily Pain Scale (no, very mild, mild, moderate, severe, very severe).^60^ This question is included in the 8-item Short Form Health Survey (SF-8) and has demonstrated validity as a single-item measure of pain severity.^58,59^ A midpoint split (no to mild vs moderate to very severe) has been used to identify more complex pain conditions,^24^ and acceptable classification performance and test–retest reliability have been reported in the HUNT cohort.^33^

In this study, moderate to severe chronic pain was defined as pain lasting ≥6 months with moderate, severe, or very severe pain in the past 4 weeks. No or mild pain was defined as no, very mild, or mild pain in the past 4 weeks, including both acute pain and chronic mild pain.

#### 2.2.2. Measures of estimated cardiorespiratory fitness

Cardiorespiratory fitness was estimated using a previously validated, sex-specific nonexercise prediction model that incorporates physical activity (PA), waist circumference (WC), resting heart rate (RHR), and age (years).^42^ The sex-specific equations were as follows:

Women:

eCRF=74.74−(0.247 × age)−(0.259 × WC)−(0.114 × RHR)+(0.198 × PA index)

Men:

eCRF=100.27−(0.296 × age)−(0.369 × WC)−(0.155 × RHR)+(0.226 × PA index)

The model predicts VO_2_peak with acceptable accuracy (R^2^ = 0.56 in women and 0.61 in men).^42^ Physical activity was assessed by self-reported exercise frequency, duration, and intensity. Participants reported: frequency (“never,” “<1/wk,” “1/wk,” “2–3/week,” “nearly daily”), duration (“<15,” “15–30,” “30–60,” “>60 minutes”), and intensity (“no sweat/heavy breath,” “heavy breath and sweat,” “to exhaustion”). Total physical activity was calculated using a previously published summary index of these items.^42^ WC was measured at the umbilicus to the nearest centimeter, and RHR was obtained from automated blood pressure measurements (mean of the second and third readings).

Estimated CRF was categorized into age- and sex-specific quintiles within 10-year age groups according to published fitness category recommendations^26^ and then merged into sex-specific quintiles. Quintiles were used in cross-sectional and longitudinal analyses of pain severity. For analyses of change in eCRF and pain severity, eCRF was dichotomized at each survey as “unfit” (lowest age- and sex-specific quintile; bottom 20%) vs “fit” (upper 4 quintiles; top 80%).^35^ Based on HUNT3 and HUNT4 status, 4 change categories were defined: remained unfit, improved (unfit → fit), declined (fit → unfit), or remained fit. These categories were used as the exposure in the longitudinal analyses of change in pain severity.

#### 2.2.3. Assessment of covariates

Somatic diseases were self-reported (myocardial infarction/angina/other heart disease, stroke/brain hemorrhage, kidney disease, diabetes, cancer, epilepsy, chronic bronchitis, asthma, ankylosing spondylitis, rheumatoid arthritis, osteoarthritis) and summarized as the number of reported diseases (0, 1, or ≥2). Anxiety and depressive symptoms were assessed using the Hospital Anxiety and Depression Scale (HADS; 14 items; past week),^63^ with a cutoff ≥8 indicating clinically relevant symptoms.^3^ Smoking status was categorized as never, former, or current, and work status (employed, yes/no) was assessed by self-report.

### 2.3. Statistical analyses

Descriptive statistics for baseline characteristics are presented as mean (standard deviation) for continuous variables and numbers (percentages) for categorical variables, separately for participants with (1) no or mild pain and (2) moderate to severe chronic pain, and stratified by sex. Binary logistic regression was used for all cross-sectional and longitudinal analyses. For longitudinal analyses, baseline pain groups were analyzed separately. Among participants with no or mild baseline pain, we estimated odds ratios (ORs) for worsening vs stable/improved pain; among those with moderate to severe chronic pain, we estimated ORs for improvement vs stable/worsening pain. ORs with 95% confidence intervals (CIs) were estimated for each eCRF quintile, using the first quintile (lowest 20%) as the reference. For analyses of changes in eCRF, those who remained in the first quintile at both time points served as the reference. ORs were also estimated per 1 metabolic equivalent (MET), with eCRF treated as a continuous variable (1 MET = eCRF/3.5 mL/kg/min).^25^ Nonlinearity was assessed by adding a quadratic term, with no evidence of nonlinearity.

In the longitudinal analyses of participants with no or mild pain at baseline, we calculated the odds of an increase in pain severity of ≥1 point on the 6-point Bodily Pain Scale (SF-8).^60^ For participants with moderate to severe chronic pain, we calculated the odds of a ≥1-point reduction on the same scale. Sensitivity analyses using a ≥2-point threshold were also conducted, because the minimally important difference is uncertain.^60^ Given heterogeneity in operational definitions of chronic pain,^51^ robustness to alternative chronic pain definitions was examined by defining chronic pain by duration alone (≥6 months) and restricting the no or mild pain group to participants without chronic mild pain. Potential selection bias was assessed by comparing baseline characteristics of included and excluded participants.

The main analyses were adjusted for age, anxiety and depressive symptoms, organ diseases, smoking status, and work status. Models were stratified by sex, except for analyses of changes in eCRF and pain severity, where women and men were combined with additional adjustment for sex. Body mass index (BMI) was not adjusted for because WC is a component of the eCRF algorithm, and adding BMI could introduce multicollinearity and overadjustment, as cautioned in previous eCRF literature.^44,47^ When analyses were stratified by pain status, baseline pain was not included as a covariate. Multicollinearity was assessed using the variance inflation factor, and no concerns were identified. Statistical significance was set at *P* < 0.05; analyses were conducted in Stata 19.5.

### 2.4. Ethical considerations

This study was approved by the Data Inspectorate and the Regional Committees for Medical and Health Research Ethics for Central Norway (REK nr. 666647). All participants provided written informed consent.

## 3. Results

### 3.1. Baseline characteristics of study participants

Table 1 presents the baseline characteristics by pain severity status and sex. Participants with no or mild pain were slightly younger than those with moderate to severe chronic pain (women: 48.4 vs 54.6 years; men: 50.6 vs 54.9 years) and had higher mean eCRF (women: 33.9 vs 31.1 mL/kg/min; men: 42.0 vs 39.1 mL/kg/min). Mean Bodily Pain Scale (SF-8) scores were 1.7 in the no or mild pain group and 4.2 in the moderate to severe chronic pain group.

**Table 1:** Baseline characteristics according to pain severity status for women and men.

Characteristics	Women		Men	
	No or mild pain*	Moderate to severe chronic pain†	No or mild pain*	Moderate to severe chronic pain†
N	9903	5869	8837	3629
Age, y	48.4 ± 14.2	54.6 ± 12.4	50.6 ± 13.7	54.9 ± 11.3
BMI (kg/m^1^)	26.1 ± 4.4	27.8 ± 5.0	27.2 ± 3.4	28.1 ± 3.8
eCRF (mL/kg/min)	33.9 ± 5.8	31.1 ± 5.4	42.0 ± 7.1	39.1 ± 6.3
eCRF (METs)	9.7 ± 1.6	8.9 ± 1.5	12.0 ± 2.0	11.2 ± 1.8
Bodily pain‡	1.7 ± 0.8	4.2 ± 0.5	1.7 ± 0.8	4.2 ± 0.5
Organ disease§				
No	7057 (73.2)	2318 (42.6)	6205 (71.7)	1642 (47.9)
One	2023 (20.9)	1938 (35.7)	1870 (21.6)	1110 (32.3)
Two or more	558 (5.7)	1177 (21.7)	579 (6.6)	678 (19.9)
HADS-D				
HADS-D <8	7818 (95.1)	4323 (88.2)	6538 (93.7)	2450 (84.5)
HADS-D ≥8	395 (4.8)	579 (11.8)	434 (6.2)	449 (15.5)
HADS-A				
HADS-A <8	7246 (88.4)	3716 (76.0)	6490 (93.1)	2386 (82.6)
HADS-A ≥8	943 (11.5)	1172 (24.0)	475 (6.8)	504 (17.4)
Smoking status				
Never	4919 (50.4)	2078 (36.2)	4278 (49.1)	1191 (33.3)
Former	2787 (28.6)	2022 (35.2)	2785 (31.9)	1520 (42.5)
Current	2041 (20.9)	1647 (28.7)	1648 (18.9)	865 (24.2)
In employment‖				
Yes	8255 (90.0)	3457 (73.3)	8255 (93.7)	2369 (79.3)
No	983 (10.0)	1258 (26.7)	555 (6.3)	619 (20.7)

Data are presented as mean and standard deviation (SD) for continuous variables and as numbers (percentages) for categorical variables.

BMI (kg/m^2^), body mass index, calculated as weight in kilograms divided by height in meters squared; eCRF (mL/kg/min), estimated cardiorespiratory fitness, calculated as oxygen consumption per kilogram of body weight per minute; MET, metabolic equivalent; HADS-D, Hospital Anxiety and Depression Scale—depression subscale; HADS-A, Hospital Anxiety and Depression Scale—anxiety subscale.

* No or mild pain during the last 4 wk.

† Pain lasting ≥6 mo and of moderate to very severe intensity during the last 4 wk.

‡ Bodily Pain Scale: 1 = no pain; 2 = very mild pain; 3 = mild pain; 4 = moderate pain; 5 = severe pain; 6 = very severe pain.

§ Based on self-report of the following: myocardial infarction, angina pectoris, other heart diseases, stroke/brain hemorrhage, kidney disease, diabetes, cancer, epilepsy, chronic bronchitis, asthma, ankylosing spondylitis, rheumatoid arthritis, or osteoarthritis.

‖ Among participants aged 20–64 (working population).

Pain severity decreased slightly across increasing eCRF quintiles in both sexes (see Table 1, supplemental digital content, http://links.lww.com/PR9/A409). Baseline characteristics of included and excluded participants are shown in supplemental digital content (see Table 2, http://links.lww.com/PR9/A409), where excluded participants had somewhat higher pain burden, more adverse lifestyle factors, and more anxiety and depressive symptoms than those retained.

### 3.2. Changes in pain severity from HUNT3 to HUNT4

Among participants with no or mild pain at baseline, 52% of women and 48% of men reported increased pain severity at follow-up. Among those with moderate to severe chronic pain, 40% of women and 45% of men reported reduced pain, with about one-third improving to no or mild pain, while a substantial proportion remained stable (see Tables 3–4, supplemental digital content, http://links.lww.com/PR9/A409). In this chronic pain group, those who improved had slightly higher baseline eCRF, fewer somatic diseases, and lower anxiety and depressive symptoms than those who did not (see Table 5, supplemental digital content, http://links.lww.com/PR9/A409).

### 3.3. Cross-sectional associations between estimated cardiorespiratory fitness and chronic pain

**Figure 2.**
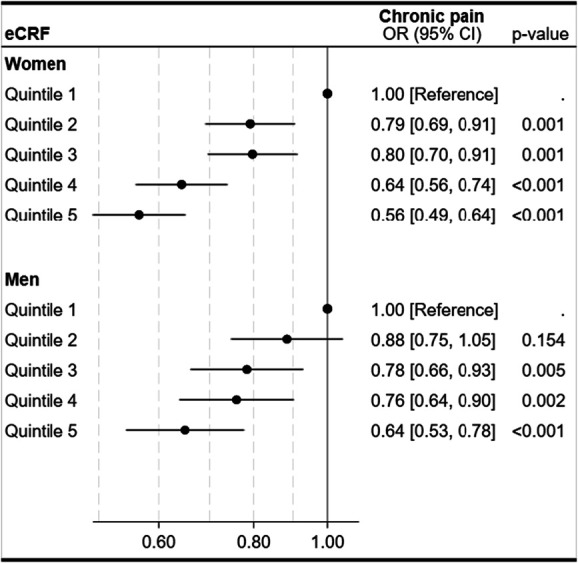
Cross-sectional associations between quintiles of estimated cardiorespiratory fitness (eCRF) and moderate to severe chronic pain, presented for women (n = 11,077) and men (n = 7769). Odds ratios (ORs) and 95% confidence intervals (CIs) are shown for each eCRF quintile, with quintile 1 as the reference. Odds ratios are adjusted for age, organ diseases (myocardial infarction, angina pectoris, other heart diseases, stroke/brain hemorrhage, kidney disease, diabetes, cancer, epilepsy, chronic bronchitis, asthma, ankylosing spondylitis, rheumatoid arthritis, or osteoarthritis), anxiety and depressive symptoms (HADS-D, HADS-A), smoking status, and work status.

Higher eCRF was associated with lower odds of moderate to severe chronic pain (Fig. 2). Compared with the first (lowest) quintile, women in the fifth quintile had 44% lower odds (OR 0.56, 95% CI 0.49–0.64) and men 36% lower odds (OR 0.64, 95% CI 0.53–0.78). Per 1-MET increase in eCRF, the OR was 0.84 (95% CI 0.81–0.87) in women and 0.89 (95% CI 0.85–0.93) in men (not shown).

### 3.4. Associations between estimated cardiorespiratory fitness and change in pain severity

**Figure 3.**
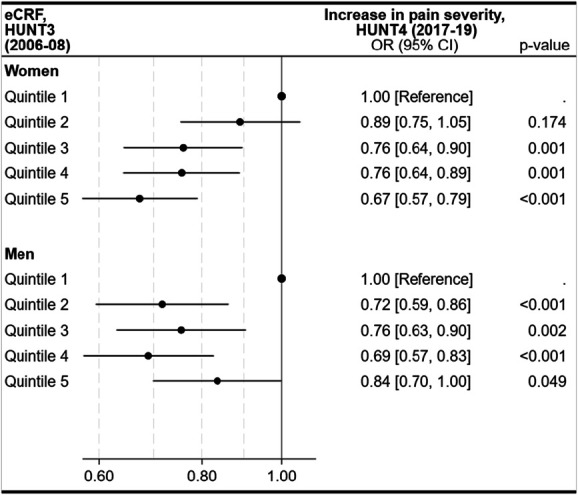
Longitudinal associations between quintiles of estimated cardiorespiratory fitness (eCRF) and change (increase) in pain severity among participants with no or mild pain at baseline (HUNT3), presented for women (n = 6463) and men (n = 5054). Odds ratios (ORs) and 95% confidence intervals (CIs) are shown for each eCRF quintile, with quintile 1 as the reference. ORs are adjusted for age, organ diseases (myocardial infarction, angina pectoris, other heart diseases, stroke/brain hemorrhage, kidney disease, diabetes, cancer, epilepsy, chronic bronchitis, asthma, ankylosing spondylitis, rheumatoid arthritis, or osteoarthritis), anxiety and depressive symptoms (HADS-D, HADS-A), smoking status, and work status.

Among participants with no or mild baseline pain, higher baseline eCRF was associated with lower odds of pain worsening over 11 years (Fig. 3). Compared with the first (lowest) quintile, women in the fifth quintile had 33% lower odds (OR 0.67, 95% CI 0.57–0.79) and men in the fourth quintile 31% lower odds (OR 0.69, 95% CI 0.57–0.83). Per 1-MET increase in eCRF, the OR for worsening pain was 0.87 (95% CI 0.84–0.91) in women and 0.96 (95% CI 0.92–1.00) in men (not shown).

**Figure 4.**
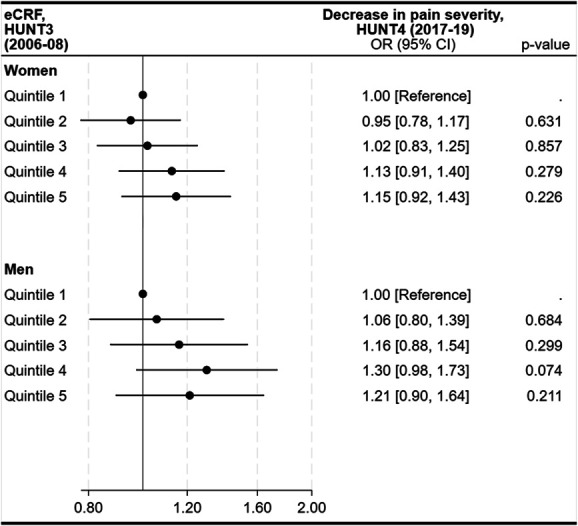
Longitudinal associations between quintiles of estimated cardiorespiratory fitness (eCRF) and change (decrease) in pain severity among participants with moderate to severe chronic pain at baseline (HUNT3), presented for women (n = 3458) and men (n = 1854). Odds ratios (ORs) and 95% confidence intervals (CIs) are shown for each eCRF quintile, with quintile 1 as the reference. OR are adjusted for age, organ diseases (myocardial infarction, angina pectoris, other heart diseases, stroke/brain hemorrhage, kidney disease, diabetes, cancer, epilepsy, chronic bronchitis, asthma, ankylosing spondylitis, rheumatoid arthritis, or osteoarthritis), anxiety and depressive symptoms (HADS-D, HADS-A), smoking status, and work status.

In participants with moderate to severe chronic pain at baseline, higher eCRF was associated with a nonsignificant trend toward reduced pain over 11 years (Fig. 4).

### 3.5. Associations between change in estimated cardiorespiratory fitness and change in pain severity

**Figure 5.**
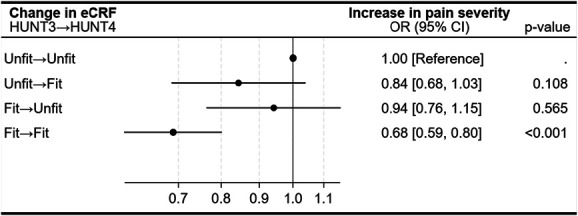
Longitudinal associations between changes in estimated cardiorespiratory fitness (eCRF) and changes (increase) in pain severity among participants with no or mild pain at baseline (n = 9647). Estimated cardiorespiratory fitness categories were defined as unfit (≤20% of participants) and fit (>20% of participants). Odds ratios (ORs) and 95% confidence intervals (CIs) are shown for each eCRF change category, with the “unfit → unfit” group as the reference. ORs are adjusted for age, sex, organ diseases (myocardial infarction, angina pectoris, other heart diseases, stroke/brain hemorrhage, kidney disease, diabetes, cancer, epilepsy, chronic bronchitis, asthma, ankylosing spondylitis, rheumatoid arthritis, or osteoarthritis), anxiety and depressive symptoms (HADS-D, HADS-A), smoking status, and work status.

In participants with no or mild baseline pain, maintained high eCRF was associated with lower odds of pain worsening (Fig. 5). Compared with those remaining in the lowest 20% of eCRF at both HUNT3 and HUNT4, participants in the upper 80% at both time points had 32% lower odds of increased pain severity (OR 0.68, 95% CI 0.59–0.80). Those who improved from unfit to fit or declined from fit to unfit had ORs of 0.84 (95% CI 0.68–1.03) and 0.94 (95% CI 0.76–1.15), respectively, but these estimates were not statistically significant.

**Figure 6.**
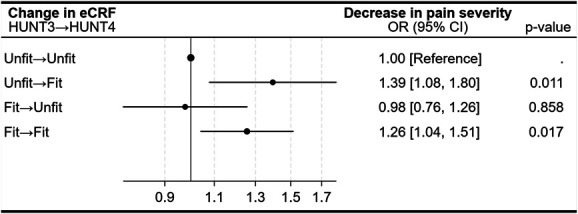
Longitudinal associations between changes in estimated cardiorespiratory fitness (eCRF) and changes (decrease) in pain severity among participants with moderate to severe chronic pain at baseline (n = 4238). eCRF categories were defined as unfit (≤20% of participants) and fit (>20% of participants). Odds ratios (ORs) and 95% confidence intervals (CIs) are shown for each eCRF change category, with the “unfit → unfit” group as the reference. ORs are adjusted for age, sex, organ diseases (myocardial infarction, angina pectoris, other heart diseases, stroke/brain hemorrhage, kidney disease, diabetes, cancer, epilepsy, chronic bronchitis, asthma, ankylosing spondylitis, rheumatoid arthritis, or osteoarthritis), anxiety and depressive symptoms (HADS-D, HADS-A), smoking status, and work status.

In participants with moderate to severe chronic pain at baseline, both maintained high and improved eCRF were associated with higher odds of reduced pain severity (Fig. 6). Compared with those remaining in the lowest 20% at both surveys, participants in the upper 80% at both time points had 26% higher odds (OR 1.26, 95% CI 1.04–1.51), and those improving from unfit at HUNT3 to fit at HUNT4 had 39% higher odds (OR 1.39, 95% CI 1.08–1.80) of reporting reduced pain severity.

Sensitivity analyses with a stricter threshold for change in pain severity (≥2 points) and alternative chronic pain definitions showed patterns similar to those observed in the longitudinal analyses (see Figs. 1–6, supplemental digital content, http://links.lww.com/PR9/A409).

## 4. Discussion

### 4.1. Main findings

This population-based study found that higher eCRF was associated with lower odds of moderate to severe chronic pain in both women and men. The association followed a clear dose–response pattern, with the lowest odds observed among the most fit participants.

Longitudinally, among participants with no or mild pain at baseline, both higher baseline eCRF and maintained high eCRF over 11 years were significantly associated with lower odds of pain worsening. In participants with moderate to severe chronic pain at baseline, higher baseline eCRF showed a nonsignificant trend toward reduced pain severity. However, those who improved or maintained high eCRF over 11 years had significantly greater odds of reporting reduced pain severity.

### 4.2. Comparisons with earlier findings

Our results are consistent with previous studies showing an inverse relationship between CRF and chronic pain severity in a variety of conditions, including primary headache disorders,^20^ low back pain,^18,43^ ankylosing spondylitis,^17^ and fibromyalgia.^15^ In contrast, some studies report no such associations in patients with low back pain,^61^ and fibromyalgia.^55^ Most earlier studies focused on specific diagnoses and included relatively small patient samples.^20–22,48–50,55,61^ By contrast, our study examined eCRF and pain severity in a large general population, providing findings that are generalizable to adults both with and without chronic pain. In population studies, chronic pain has more often been studied in relation to physical activity, which is strongly correlated with CRF.^31,41^ Similar to our findings, Fjeld et al. and Grasdalsmoen et al. reported a dose–response relationship, where higher levels of physical activity were associated with less chronic pain.^14,17^ Landmark et al. observed a similar association among older adults,^32^ but a U-shaped pattern in younger and middle-aged adults. Notably, exercise intensity in their study was generally associated with less chronic pain,^32^ which is relevant to our findings, as exercise intensity is a key driver of improvements in CRF.^26^ In longitudinal population studies, Årnes et al. reported higher pain tolerance with higher and increasing activity levels.^64^ Likewise, we observed that participants who improved or maintained high eCRF had the most favorable pain outcomes. A population-based study reported that baseline physical inactivity was associated with a higher prevalence of widespread chronic pain 11 years later,^23^ but baseline pain was not accounted for. In our study, participants were stratified by baseline pain status, making additional adjustment for baseline pain unnecessary. In another HUNT study, moderate or higher exercise levels were associated with less pain over 12 months; however, after adjustment for baseline pain, the association persisted only in men.^34^ By contrast, our study spans 11 years, providing insight into the long-term relationship between eCRF and pain severity. Despite this extended interval between HUNT3 and HUNT4, most participants with chronic pain reported stable pain levels, consistent with earlier population research.^33^ Even so, fluctuations in pain and fitness during follow-up cannot be discounted, and previous studies show that the physical activity–pain association can shift rapidly within individuals.^5,34^ Future studies should therefore assess eCRF and pain at shorter, repeated intervals to better capture this dynamic interplay.

### 4.3. Possible mechanisms

Several mechanisms may influence the observed associations between eCRF and pain severity. One potential explanation is that physical activity may modulate nociplastic pain mechanisms in the central nervous system.^6^ Nociplastic pain is characterized by altered nociceptive processing, with reduced endogenous pain inhibition and increased pain facilitation.^13,28^ A recent systematic review found no overall effect of exercise on markers of central sensitization, but reported beneficial effects of motor control exercises in chronic neck pain, suggesting that some exercise modalities may help restore central pain regulation in selected conditions.^6^ Behavioral adaptations may also be important. Active individuals with higher fitness levels generally show greater pain tolerance than inactive individuals,^64^ and those with higher eCRF are likely to have more experience managing discomfort through systematic training. This may enhance their ability to cope with pain in daily life and may contribute to lower reported pain levels. Systemic inflammation is another potential pathway. Elevated levels of inflammatory biomarkers such as C-reactive protein (CRP), interleukin-6 (IL-6), and interleukin-1β (IL-1β) are common in chronic pain conditions,^2,18,27,30,37,57^ and lower CRF has been associated with higher concentrations of these markers, although this relationship may be influenced by BMI.^38,62^ Whether inflammatory processes mediate the association between eCRF and pain severity remains unclear. Exercise-induced hypoalgesia may also be relevant, although it likely reflects a more acute mechanism. Exercise-induced hypoalgesia refers to a transient reduction in pain sensitivity after exercise, particularly following high-intensity exercise.^45,46^ It is mediated by central and peripheral mechanisms, including the release of beta-endorphins and other pain-modulating substances,^4,40^ but typically subsides within about 30 minutes.^45^ Thus, while exercise-induced hypoalgesia may contribute to short-term pain relief, its relevance for long-term modulation of chronic pain remains uncertain.^40^

### 4.4. Methodological strengths and limitations

This study has several strengths. The longitudinal design with repeated measures of pain severity and eCRF, complemented by cross-sectional analyses in the same cohort, allowed us to assess both changes over time and point-in-time associations. The large sample size enabled longitudinal analyses in 2 pain subgroups and sex-specific analyses in most cases. Access to a broad set of potential confounders, together with acceptable participation rates in HUNT3 (51.1%) and HUNT4 (54%), likely reduced bias and strengthened the validity of the findings.

The study also has limitations. The Bodily Pain Scale (SF-8) is a reliable and valid measure of pain severity,^58,59^ but there is no consensus on what constitutes a clinically meaningful change. We defined change in pain severity as ≥1 point on the 6-point scale and conducted sensitivity analyses using a ≥2-point threshold, which yielded similar trends in both groups. Compared with a more commonly used 10-point numerical rating scale, this would be closer to a moderate change, often defined as 30% reduction, than a minimally clinical change, often defined as a 1-point change.^9^ However, the ordinal categories may not represent equal perceived differences (eg, the step from “no pain” to “very mild pain” may be smaller than from “moderate” to “severe”), and with only 6 response categories, the SF-8 may be more prone to floor and ceiling effects and less sensitive to change than continuous instruments such as a 0 to 10 NRS making it difficult to detect small but potentially important changes in pain.^7,60^ Another limitation is the potential selection of individuals with more favorable psychological and behavioral profiles into the no or mild pain group. People who tolerate discomfort better and tend to remain physically active may both report lower pain and maintain higher eCRF, and such unmeasured traits could partly account for the observed associations between eCRF and pain severity. Moreover, because the nonexercise eCRF algorithm includes physical activity measures, our exposure reflects a combination of activity behavior and physiological fitness. Physical activity is closely associated with pain,^14,17,23,32^ and part of the observed association between eCRF and pain severity may therefore reflect differences in habitual activity patterns rather than fitness per se. Physical activity in the eCRF algorithm was self-reported and may have led to overestimation of activity levels,^10^ potentially biasing eCRF upward and weakening associations. However, most associations remained statistically significant, suggesting that these limitations did not substantially alter the overall findings. It is also important to note that our figures display categorical odds ratios rather than continuous exposure plots or absolute risks, which may obscure finer details of the underlying associations. Another limitation is attrition and exclusion due to missing data, which likely resulted in a somewhat healthier analytical sample, potentially biasing associations towards the null and affecting internal validity. Finally, the relative ethnic and genetic homogeneity of the HUNT population may limit the generalizability to more diverse populations.

### 4.5. Conclusion

In this population-based study, higher eCRF was associated with lower odds of chronic pain, with the lowest odds observed among the most fit participants. Among participants with no or mild pain at baseline, higher and persistently high eCRF were associated with lower odds of pain worsening, whereas among those with chronic pain, increasing or maintaining high eCRF was associated with greater odds of pain reduction. Overall, higher eCRF was associated with more favorable pain outcomes; however, these observational findings do not permit causal inference.

## Disclosures

The authors have no conflict of interest to declare.

## Supplemental digital content

Supplemental digital content associated with this article can be found online at http://links.lww.com/PR9/A409.

## References

[1] Classification of chronic pain: descriptions of chronic pain syndromes and definitions of pain terms. PAIN 1986;3:226. 3461421

[2] Abdelhafiz D Baker T Glascow DA Abdelhafiz A. Biomarkers for the diagnosis and treatment of rheumatoid arthritis—a systematic review. Postgrad Med 2023;135:214–23. 35275765 10.1080/00325481.2022.2052626

[3] Bjelland I Dahl AA Haug TT Neckelmann D. The validity of the hospital anxiety and depression scale: an updated literature review. J Psychosomatic Res 2002;52:69–77. 10.1016/s0022-3999(01)00296-3 11832252

[4] Borisovskaya A Chmelik E Karnik A. Exercise and chronic pain. In: Xiao J, editor. Physical Exercise for Human Health. Singapore: Springer Nature Singapore, 2020. pp. 233–53. 10.1007/978-981-15-1792-1_16 32342462

[5] Carbonell-Baeza A Ruiz JR Aparicio VA Ortega FB Delgado-Fernández M. The 6-minute walk test in female fibromyalgia patients: relationship with tenderness, symptomatology, quality of life, and coping strategies. Pain Manag Nurs 2013;14:193–9. 24315242 10.1016/j.pmn.2011.01.002

[6] Chen KK Rolan P Hutchinson MR Dickson C de Zoete RMJ. Exercise-induced changes in central sensitization outcomes in individuals with chronic musculoskeletal pain: a systematic review with meta-analysis. Eur J Pain 2024;28:1431–49. 38662515 10.1002/ejp.2277

[7] Chien CW Bagraith KS Khan A Deen M Strong J. Comparative responsiveness of verbal and numerical rating scales to measure pain intensity in patients with chronic pain. J Pain 2013;14:1653–62. 24290445 10.1016/j.jpain.2013.08.006

[8] Dominick CH Blyth FM Nicholas MK. Unpacking the burden: understanding the relationships between chronic pain and comorbidity in the general population. PAIN 2012;153:293–304. 22071318 10.1016/j.pain.2011.09.018

[9] Dworkin RH Turk DC McDermott MP Peirce-Sandner S Burke LB Cowan P Farrar JT Hertz S Raja SN Rappaport BA Rauschkolb C Sampaio C. Interpreting the clinical importance of group differences in chronic pain clinical trials: IMMPACT recommendations. PAIN 2009;146:238–44. 19836888 10.1016/j.pain.2009.08.019

[10] Dyrstad SM Hansen BH Holme IM Anderssen SA. Comparison of self-reported versus accelerometer-measured physical activity. Med Sci Sports Exerc 2014;46:99–106. 23793232 10.1249/MSS.0b013e3182a0595f

[11] Eriksen J Jensen MK Sjøgren P Ekholm O Rasmussen NK. Epidemiology of chronic non-malignant pain in Denmark. PAIN 2003;106:221–8. 14659505 10.1016/S0304-3959(03)00225-2

[12] Eriksen J Sjøgren P Ekholm O Rasmussen NK. Health care utilisation among individuals reporting long-term pain: an epidemiological study based on Danish National Health Surveys. Eur J Pain 2004;8:517–23. 15531219 10.1016/j.ejpain.2003.12.001

[13] Fitzcharles M-A Cohen SP Clauw DJ Littlejohn G Usui C Häuser W. Nociplastic pain: towards an understanding of prevalent pain conditions. Lancet 2021;397:2098–110. 34062144 10.1016/S0140-6736(21)00392-5

[14] Fjeld MK Årnes AP Engdahl B Morseth B Hopstock LA Horsch A Stubhaug A Strand BH Nielsen CS Steingrímsdóttir ÓA. Consistent pattern between physical activity measures and chronic pain levels: the Tromsø Study 2015 to 2016. PAIN 2023;164:838–47. 36083173 10.1097/j.pain.0000000000002773 PMC10026831

[15] Gaudreault N Boulay P. Cardiorespiratory fitness among adults with fibromyalgia. Breathe 2018;14:e25–e33. 30131831 10.1183/20734735.019717 PMC6095234

[16] GBD 2016 Disease and Injury Incidence and Prevalence Collaborators. Global, regional, and national incidence, prevalence, and years lived with disability for 328 diseases and injuries for 195 countries, 1990-2016: a systematic analysis for the global burden of Disease study 2016. Lancet 2017;390:1211–59. 28919117 10.1016/S0140-6736(17)32154-2 PMC5605509

[17] Grasdalsmoen M Engdahl B Fjeld MK Steingrímsdóttir ÓA Nielsen CS Eriksen HR Lønning KJ Sivertsen B. Physical exercise and chronic pain in university students. PLoS One 2020;15:e0235419. 32589694 10.1371/journal.pone.0235419 PMC7319292

[18] Groven N Fors EA Reitan SK. Patients with fibromyalgia and chronic fatigue syndrome show increased hsCRP compared to healthy controls. Brain Behav Immun 2019;81:172–7. 31176728 10.1016/j.bbi.2019.06.010

[19] Hadi MA McHugh GA Closs SJ. Impact of chronic pain on patients' quality of life: a comparative mixed-methods study. J Patient Experience 2019;6:133–41. 10.1177/2374373518786013 PMC6558939 31218259

[20] Hagen K Wisløff U Ellingsen Ø Stovner LJ Linde M. Headache and peak oxygen uptake: the HUNT3 study. Cephalalgia 2016;36:437–44. 26207022 10.1177/0333102415597528

[21] Halvorsen S Vøllestad NK Fongen C Provan SA Semb AG Hagen KB Dagfinrud H. Physical fitness in patients with ankylosing spondylitis: comparison with population controls. Phys Ther 2012;92:298–309. 22095208 10.2522/ptj.20110137

[22] Heneweer H Picavet HSJ Staes F Kiers H Vanhees L. Physical fitness, rather than self-reported physical activities, is more strongly associated with low back pain: evidence from a working population. Eur Spine J 2012;21:1265–72. 22134487 10.1007/s00586-011-2097-7 PMC3389121

[23] Holth HS Werpen HK Zwart JA Hagen K. Physical inactivity is associated with chronic musculoskeletal complaints 11 years later: results from the Nord-Trøndelag health study. BMC Musculoskelet Disord 2008;9:159. 19046448 10.1186/1471-2474-9-159 PMC2606680

[24] Jensen MK Sjøgren P Ekholm O Rasmussen NK Eriksen J. Identifying a long-term/chronic, non-cancer pain population using a one-dimensional verbal pain rating scale: an epidemiological study. Eur J Pain 2004;8:145–52. 14987624 10.1016/S1090-3801(03)00088-0

[25] Jetté M Sidney K Blümchen G. Metabolic equivalents (METS) in exercise testing, exercise prescription, and evaluation of functional capacity. Clin Cardiol 1990;13:555–65. 2204507 10.1002/clc.4960130809

[26] Karlsen T Aamot I-L Haykowsky M Rognmo Ø. High intensity interval training for maximizing health outcomes. Prog Cardiovasc Dis 2017;60:67–77. 28385556 10.1016/j.pcad.2017.03.006

[27] Koch A Zacharowski K Boehm O Stevens M Lipfert P von Giesen HJ Wolf A Freynhagen R. Nitric oxide and pro-inflammatory cytokines correlate with pain intensity in chronic pain patients. Inflamm Res 2007;56:32–7. 17334668 10.1007/s00011-007-6088-4

[28] Kosek E Cohen M Baron R Gebhart GF Mico J-A Rice ASC Rief W Sluka AK. Do we need a third mechanistic descriptor for chronic pain states? PAIN 2016;157:1382–6. 26835783 10.1097/j.pain.0000000000000507

[29] Krokstad S Langhammer A Hveem K Holmen TL Midthjell K Stene TR Bratberg G Heggland J Holmen J. Cohort profile: the HUNT Study, Norway. Int J Epidemiol 2013;42:968–77. 22879362 10.1093/ije/dys095

[30] Kumbhare D Hassan S Diep D Duarte FCK Hung J Damodara S West DWD Selvaganapathy PR. Potential role of blood biomarkers in patients with fibromyalgia: a systematic review with meta-analysis. PAIN 2022;163:1232–53. 34966131 10.1097/j.pain.0000000000002510

[31] Kurtze N Rangul V Hustvedt B-E Flanders WD. Reliability and validity of self-reported physical activity in the Nord-Trøndelag Health Study—HUNT 1. Scand J Public Health 2008;36:52–61. 18426785 10.1177/1403494807085373

[32] Landmark T Romundstad P Borchgrevink PC Kaasa S Dale O. Associations between recreational exercise and chronic pain in the general population: evidence from the HUNT 3 study. PAIN 2011;152:2241–7. 21601986 10.1016/j.pain.2011.04.029

[33] Landmark T Romundstad P Dale O Borchgrevink PC Kaasa S. Estimating the prevalence of chronic pain: validation of recall against longitudinal reporting (the HUNT pain study). PAIN 2012;153:1368–73. 22575226 10.1016/j.pain.2012.02.004

[34] Landmark T Romundstad PR Borchgrevink PC Kaasa S Dale O. Longitudinal associations between exercise and pain in the general population: the HUNT Pain Study. PLOS One 2013;8:e65279. 23776464 10.1371/journal.pone.0065279 PMC3680414

[35] Lee D-C Sui X Ortega FB Kim Y-S Church TS Winett RA Ekelund U Katzmarzyk PT Blair SN. Comparisons of leisure-time physical activity and cardiorespiratory fitness as predictors of all-cause mortality in men and women. Br J Sports Med 2011;45:504–10. 20418526 10.1136/bjsm.2009.066209

[36] Lestoquoy AS Laird LD Mitchell S Gergen-Barnett K Negash NL McCue K Enad R Gardiner P. Living with chronic pain: evaluating patient experiences with a medical group visit focused on mindfulness and non-pharmacological strategies. Complement Therapies Med 2017;35:33–8. 10.1016/j.ctim.2017.09.002 29154064

[37] Lim YZ Wang Y Cicuttini FM Hughes HJ Chou L Urquhart DM Ong PX Hussain SM. Association between inflammatory biomarkers and nonspecific low back pain: a systematic review. Clin J Pain 2020;36:379–89. 31990692 10.1097/AJP.0000000000000810

[38] Madssen E Skaug E-A Wisløff U Ellingsen Ø Videm V. Inflammation is strongly associated with cardiorespiratory fitness, sex, BMI, and the metabolic syndrome in a self-reported healthy population: HUNT3 Fitness Study. Mayo Clinic Proc 2019;94:803–10. 10.1016/j.mayocp.2018.08.040 30935704

[39] Myers J McAuley P Lavie CJ Despres JP Arena R Kokkinos P. Physical activity and cardiorespiratory fitness as major markers of cardiovascular risk: their independent and interwoven importance to health status. Prog Cardiovasc Dis 2015;57:306–14. 25269064 10.1016/j.pcad.2014.09.011

[40] Naugle KM Fillingim RB Riley JL III. A meta-analytic review of the hypoalgesic effects of exercise. J Pain 2012;13:1139–50. 23141188 10.1016/j.jpain.2012.09.006 PMC3578581

[41] Nes BM Janszky I Aspenes ST Bertheussen GF Vatten LJ Wisløff U. Exercise patterns and peak oxygen uptake in a healthy population: the HUNT study. Med Sci Sports Exerc 2012;44:1881–9. 22525768 10.1249/MSS.0b013e318258b443

[42] Nes BM Janszky I Vatten LJ Nilsen TIL Aspenes ST Wisløff U. Estimating VO_2_peak from a nonexercise prediction model: the HUNT Study, Norway. Med Sci Sports Exerc 2011;43:2024–30. 21502897 10.1249/MSS.0b013e31821d3f6f

[43] Nes BM Vatten LJ Nauman J Janszky I Wisløff U. A simple nonexercise model of cardiorespiratory fitness predicts long-term mortality. Med Sci Sports Exerc 2014;46:1159–65. 24576863 10.1249/MSS.0000000000000219

[44] Nystøyl BTS Letnes JM Nes BM Slagsvold KH Wisløff U Wahba A. Cardiorespiratory fitness and the incidence of surgery for aortic valve stenosis—the HUNT study. Eur J Cardiothoracic Surg 2023;64:ezad322. 10.1093/ejcts/ezad322 PMC10634520 37725362

[45] Rice D Nijs J Kosek E Wideman T Hasenbring MI Koltyn K Graven-Nielsen T Polli A. Exercise-induced hypoalgesia in pain-free and chronic pain populations: state of the art and future directions. J Pain 2019;20:1249–66. 30904519 10.1016/j.jpain.2019.03.005

[46] Schwarz L Kindermann W. Changes in beta-endorphin levels in response to aerobic and anaerobic exercise. Sports Med 1992;13:25–36. 1553453 10.2165/00007256-199213010-00003

[47] Sloan RA. Estimated cardiorespiratory fitness and metabolic risks. Int J Environ Res Public Health 2024;21:635. 38791849 10.3390/ijerph21050635 PMC11120962

[48] Smeets RJEM Wittink H Hidding A Knottnerus JA. Do patients with chronic low back pain have a lower level of aerobic fitness than healthy controls?: are pain, disability, fear of injury, working status, or level of leisure time activity associated with the difference in aerobic fitness level? Spine 2006;31:90–7. 16395183 10.1097/01.brs.0000192641.22003.83

[49] Soriano-Maldonado A Ortega FB Munguía-Izquierdo D. Association of cardiorespiratory fitness with pressure pain sensitivity and clinical pain in women with fibromyalgia. Rheumatol Int 2015;35:899–904. 25549601 10.1007/s00296-014-3203-z

[50] Soriano-Maldonado A Ruiz JR Aparicio VA Estévez-López F Segura-Jiménez V Álvarez-Gallardo IC Carbonell-Baeza A Delgado-Fernández M Ortega FB. Association of physical fitness with pain in women with fibromyalgia: the al-Ándalus project. Arthritis Care Res 2015;67:1561–70. 10.1002/acr.22610 25939406

[51] Steingrímsdóttir ÓA Landmark T Macfarlane GJ Nielsen CS. Defining chronic pain in epidemiological studies: a systematic review and meta-analysis. PAIN 2017;158:2092–107. 28767506 10.1097/j.pain.0000000000001009

[52] Stewart WF Ricci JA Chee E Morganstein D Lipton R. Lost productive time and cost due to common pain conditions in the US workforce. JAMA 2003;290:2443–54. 14612481 10.1001/jama.290.18.2443

[53] Treede RD Rief W Barke A Aziz Q Bennett MI Benoliel R Cohen M Evers S Finnerup NB First MB Giamberardino MA Kaasa S Korwisi B Kosek E Lavand'homme P Nicholas M Perrot S Scholz J Schug S Smith BH Svensson P Vlaeyen JWS Wang SJ. Chronic pain as a symptom or a disease: the IASP classification of chronic pain for the International Classification of Diseases (ICD-11). PAIN 2019;160:19–27. 30586067 10.1097/j.pain.0000000000001384

[54] Treede RD Rief W Barke A Aziz Q Bennett MI Benoliel R Cohen M Evers S Finnerup NB First MB Giamberardino MA Kaasa S Kosek E Lavand'homme P Nicholas M Perrot S Scholz J Schug S Smith BH Svensson P Vlaeyen JWS Wang SJ. A classification of chronic pain for ICD-11. PAIN 2015;156:1003–7. 25844555 10.1097/j.pain.0000000000000160 PMC4450869

[55] Valim V Oliveira L Suda A Silva L de Assis M Barros Neto T Feldman D Natour J. Aerobic fitness effects in fibromyalgia. J Rheumatol 2003;30:1060–9. 12734907

[56] Vanhees L Lefevre J Philippaerts R Martens M Huygens W Troosters T Beunen G. How to assess physical activity? How to assess physical fitness? Eur J Cardiovasc Prev Rehabil 2005;12:102–14. 15785295 10.1097/01.hjr.0000161551.73095.9c

[57] Vedova CD Cathcart S Dohnalek A Lee V Hutchinson MR Immink MA Hayball J. Peripheral Interleukin-1β levels are elevated in chronic tension-type headache patients. Pain Res Manag 2013;18:301–6. 23957020 10.1155/2013/796161 PMC3917793

[58] Von Korff M Jensen MP Karoly P. Assessing global pain severity by self-report in clinical and health services research. Spine 2000;25:3140–51. 11124730 10.1097/00007632-200012150-00009

[59] Ware JE Kosinski M Dewey JE Gandek B. How to score and interpret single-item health status measures: a manual for users of the SF-8 health survey. QualityMetric Incorporated 2001;15:5.

[60] Williamson A Hoggart B. Pain: a review of three commonly used pain rating scales. J Clin Nurs 2005;14:798–804. 16000093 10.1111/j.1365-2702.2005.01121.x

[61] Wittink H Michel TH Sukiennik A Gascon C Rogers W. The association of pain with aerobic fitness in patients with chronic low back pain. Arch Phys Med Rehabil 2002;83:1467–71. 12370889 10.1053/apmr.2002.34597

[62] Zdziarski LA Wasser JG Vincent HK. Chronic pain management in the obese patient: a focused review of key challenges and potential exercise solutions. J Pain Res 2015;8:63–77. 25709495 10.2147/JPR.S55360 PMC4332294

[63] Zigmond AS Snaith RP. The hospital anxiety and depression scale. Acta Psychiatr Scand 1983;67:361–70. 6880820 10.1111/j.1600-0447.1983.tb09716.x

[64] Årnes AP Nielsen CS Stubhaug A Fjeld MK Johansen A Morseth B Strand BH Wilsgaard T Steingrímsdóttir ÓA. Longitudinal relationships between habitual physical activity and pain tolerance in the general population. PLoS One 2023;18:e0285041. 37224163 10.1371/journal.pone.0285041 PMC10208467

[65] Åsvold BO Langhammer A Rehn TA Kjelvik G Grøntvedt TV Sørgjerd EP Fenstad JS Heggland J Holmen O Stuifbergen MC Vikjord SAA Brumpton BM Skjellegrind HK Thingstad P Sund ER Selbæk G Mork PJ Rangul V Hveem K Næss M Krokstad S. Cohort profile update: the HUNT study, Norway. Int J Epidemiol 2022;52:e80–e91. 10.1093/ije/dyac095 PMC9908054 35578897

