# Supplementary material for: Cardiorespiratory fitness and pain severity: Longitudinal associations from the general population—The HUNT study
Source: Pain Rep. 2026 Apr 28;11(3):e1447. doi: 10.1097/PR9.0000000000001447 (PMC13132283; doi:10.1097/PR9.0000000000001447)
Supplement: Supplementary file 1 [file painreports-11-e1447-s001.pdf]

Supplementary material

Supplementary Table 1: **Baseline characteristics according to levels of estimated cardiorespiratory fitness for women and men, stratified by age- and sex-specific quintiles.**

	Women					Men				
**Characteristics**	**1st Quintile**	**2ndQuintile**	**3rd Quintile**	**4th Quintile**	**5th Quintile**	**1st Quintile**	**2ndQuintile**	**3rd Quintile**	**4th Quintile**	**5th Quintile**
**No. participants**	3067	3067	3063	3066	3062	2158	2154	2155	2153	2151
**Age in years**	51 ± 13.5	51 ± 13.4	50 ± 13.3	50 ± 13.3	49 ± 13.3	53 ± 13.1	53 ± 13.1	52 ± 13.1	52 ± 13.2	51 ± 13.4
**BMI (kg/m^2)**	31.6 ± 4.7	27.5 ± 3.4	25.9 ± 3.1	24.7 ± 2.9	23 ± 2.5	30.9 ± 3.5	28.1 ± 2.6	26.9 ± 2.5	25.8 ± 2.3	24.6 ± 2.2
**eCRF (ml/kg^1/min^1)**	27.0 ± 4.4	30.8 ± 4.2	33.0 ± 4.2	35.4 ± 4.2	38.5 ± 4.5	33.9 ± 5.3	38.5 ± 4.9	41.2 ± 4.9	43.7 ± 5.0	47.9 ± 5.6
**eCRF (METs)**	7.7 ± 1.2	8.8 ± 1.2	9.4 ± 1.2	10.1 ± 1.2	11.0 ± 1.2	9.6 ± 1.5	11.0 ± 1.4	11.7 ± 1.4	12.5 ± 1.4	13.7 ± 1.6
**Bodily pain** [a)]	3.1 ± 1.4	2.9 ± 1.4	2.8 ± 1.4	2.6 ± 1.4	2.5 ± 1.4	2.8 ± 1.4	2.6 ± 1.4	2.5 ± 1.3	2.5 ± 1.4	2.3 ± 1.3
**Organ disease** [b)]										
No	1607 (55.4)	1814 (61.9)	1891 (64.4)	2056 (69.6)	2058 (70.0)	1170 (56.9)	1361 (65.2)	1379 (65.7)	1420 (67.8)	1443 (68.7)
One	831 (28.6)	805 (27.4)	775 (26.4)	686 (23.2)	6773 (22.8)	5998 (29.1)	512 (24.5)	489 (23.3)	491 (23.4)	502 (23.9
Two or more	460 (16.0)	316 (10.7	267 (9.2)	212 (7.2)	215 (7.2)	286 (14.0)	214 (10.3)	231 (11.0)	184 (8.8)	157 (7.4)
**HADS-D**										
HADS-D <8	2322 (90.8)	2371 (93.0)	2416 (94.6)	2405 (93.3)	2505 (95.3)	1581 (89.8)	1555 (91.2)	1622 (91.9)	1613 (92.3)	1670 (94.9)
HADS-D ≥8	236 (9.2)	181 (7.0)	139 (5.4)	171 (6.7)	124 (4.7)	179 (10.2)	149 (8.8)	143 (8.1)	134 (7.7)	91 (5.1)
**HADS-A**										
HADS-A <8	2117 (82.5)	2171 (85.0)	2149 (84.5)	2181 (84.9)	2242 (85.4)	1566 (89.5)	1537 (90.1)	1578 (90.3)	1594 (90.8)	1610 (91.7)
HADS-A ≥8	450 (17.5)	383 (15.0)	393 (15.5)	387 (15.1)	382 (14.6)	168 (10.5)	168 (9.9)	169 (9.7)	161 (9.2)	145 (8.3)
**Smoking status**										
Never	1346 (44.7)	1304 (43.4)	1359 (45.1)	1425 (47.1)	1547 (51.3)	814 (38.2)	934 (43.9)	960 (44.9)	1054 (49.4)	1212 (57.0)
Former	974 (32.3)	962 (31.8)	975 (32.4)	1016 (33.6)	926 (30.7)	903 (42.3)	796 (37.5)	770 (36.1)	733 (34.4)	639 (30.0)
Current	690 (23.0)	757 (25.0)	689 (22.5)	582 (19.3)	541 (18.0)	416 (19.5)	394 (18.6)	406 (19.0)	346 (16.2)	270 (13.0)
**In employment** [c)]										
Yes	1995 (79.0)	2175 (84.6)	2198 (84.6)	2296 (87.4)	2323 (86.8)	1472 (85.4)	1549 (88.4)	1599 (90.6)	1584 (89.3)	1624 (90.7)
No	530 (21.0)	396 (15.4)	400 (15.4)	332 (12.6)	352 (13.2)	252 (14.6)	204 (11.6)	166 (9.4)	190 (10.7)	166 (9.3)

Data are presented as mean and standard deviation (SD) for continuous variables and as numbers (percentages) for categorical variables. The first quintile represents the 20% of participants with the lowest estimated cardiorespiratory fitness (eCRF) within each age- and sex- specific group. Abbreviations: BMI (kg/m²) = body mass index, calculated as weight in kilograms divided by height in meters squared; eCRF (ml/kg/min) = estimated cardiorespiratory fitness, calculated as oxygen consumption per kilogram of body weight per minute; MET = metabolic equivalent; HADS-D = Hospital Anxiety and Depression Scale – Depression subscale; HADS-A = Hospital Anxiety and Depression Scale – Anxiety subscale. Footnotes: [a)] *Bodily Pain Scale: 1=no pain; 2=very mild pain; 3=mild pain; 4=moderate pain; 5=severe pain; 6=very severe pain.* [b)] *Based on self-report of the following: myocardial infarction, angina pectoris, other heart diseases, stroke/brain hemorrhage, kidney disease, diabetes, cancer, epilepsy, chronic bronchitis, asthma, ankylosing spondylitis, rheumatoid arthritis, or osteoarthritis.* [c)] *Among participants aged 20–64 years (working population).*

Supplementary Table 2: **Baseline characteristics of participants included in the cross-sectional sample and those excluded due to missing data, HUNT3 (2006–08).**

Characteristics	Included participants	Excluded due to missing data
**N**	**18 837**	**15 070**
**Age in years**	51.5 (13.0)	51.3 (14.5)
**BMI (kg/m²)**	26.9 (4.1)	27.4 (4.4)
**eCRF (ml/kg/min)**	36.3 (7.3)	36.0 (8.1)
**Bodily pain**[a)]	2.7 (1.4)	2.8 (1.4)
**Sex**		
Women	11 083 (58.8%)	7 921 (52.6%)
Men	7 760 (41.2%)	7 143 (47.4%)
**Chronic pain** [b)]		
No	13 403 (71.1%)	8 244 (67.0%)
Yes	5 440 (28.9%)	4 058 (33.0%)
**Organ disease** [c)]		
No	11 983 (63.6%)	8 735 (64.7%)
One	4 843 (25.7%)	3 372 (25.0%)
Two or more	2 017 (10.7%)	1 386 (10.3%)
**HADS-D**		
HADS-D <8	17 548 (93.1%)	7 700 (89.0%)
HADS-D ≥8	1 295 (6.9%)	947 (11.0%)
**HADS-A**		
HADS-A <8	16 438 (87.2%)	7 259 (84.6%)
HADS-A ≥8	2 405 (12.8%)	1 325 (15.4%)
**Smoking status**		
Never	8 818 (46.8%)	6 036 (42.3%)
Former	6 448 (34.2%)	4 420 (31.0%)
Current	3 577 (19.0%)	3 812 (26.7%)
**In employment** [d)]		
Yes	16 706 (88.7%)	13 205 (88.3%)
No	2 137 (11.3%)	1 746 (11.7%)

Data are presented as mean and standard deviation (SD) for continuous variables and as numbers (percentages) for categorical variables. Abbreviations: BMI (kg/m²) = body mass index, calculated as weight in kilograms divided by height in meters squared; eCRF (ml/kg/min) = estimated cardiorespiratory fitness, calculated as oxygen consumption per kilogram of body weight per minute; HADS-D = Hospital Anxiety and Depression Scale – Depression subscale; HADS-A = Hospital Anxiety and Depression Scale – Anxiety subscale. Footnotes: [a)] Bodily Pain Scale: 1 = no pain; 2=very mild pain; 3=mild pain; 4=moderate pain; 5=severe pain; 6=very severe pain. [b)] Pain lasting ≥ 6 months and of moderate to very severe intensity during the last 4 weeks. [c)] Based on self-report of the following: myocardial infarction, angina pectoris, other heart diseases, stroke/brain hemorrhage, kidney disease, diabetes, cancer, epilepsy, chronic bronchitis, asthma, ankylosing spondylitis, rheumatoid arthritis, or osteoarthritis. [d)] Among participants aged 20 – 64 (working population).

***Supplementary Table 3:* Changes in pain severity between HUNT3 (2006-08) and HUNT4 (2017-19) among participants with no or mild pain at baseline (n = 19 755).**

	Women N (%)	Men N (%)
**Reduction in pain (total)**	**1261 (13)**	**1269 (15)**
By 1 point	909 (9)	864 (10)
By 2 points	352 (4)	405 (5)
**No change in pain**	**3309 (35)**	**3174 (37)**
**Increase in pain (total)**	**5007 (52)**	**4195 (48)**
By 1 point	2312 (24)	1998 (23)
By 2 points	1395 (15)	1217 (14)
By 3 points	1052 (11)	786 (9)
By 4 points	215 (2)	164 (2)
By 5 points	33 (<1)	30 (<1)
**Change in pain status to moderate or severe pain**	**2777 (29)**	**2090 (24)**

Values represent changes in points on the Bodily pain Scale (SF-8): 1= no pain; 2 = very mild pain; 3 = mild pain; 4 = moderate pain; 5 = severe pain; 6 = very severe pain.

***Supplementary Table 4*: Changes in pain severity between HUNT3 (2006-08) and HUNT4 (2017-19) among participants with moderate to severe chronic pain at baseline (n = 9254).**

	Women N (%)	Men N (%)
**Reduction in pain (total)**	**2277 (40)**	**1642 (45)**
By 1 point	1296 (22)	820 (23)
By 2 points	502 (9)	438 (12)
By 3 points	433 (8)	333 (9)
By 4 points	44 (1)	48 (1)
By 5 points	2 (<1)	3 (<1)
**No change in pain**	**2626 (46)**	**1478 (42)**
**Increase in pain (total)**	**789 (14)**	**433 (13)**
By 1 point	738 (13)	407 (12)
By 2 points	60 (1)	26 (1)
**Change in chronic pain status to no or mild pain**	**1670 (29)**	**1276 (36)**

Values represent changes in points in the Bodily Pain Scale (SF-8): 1= no pain; 2 = very mild pain; 3 = mild pain; 4 = moderate pain; 5 = severe pain; 6 = very severe pain.

Supplementary Table 5. **Baseline (HUNT3, 2006–08) characteristics of participants with moderate to severe chronic pain, according to improvement in pain severity in HUNT4 (2017–19).**

**HUNT3 (2006–08)**	**HUNT4 (2017–19)**	
**Baseline characteristics**	**No improvement [a)] (n =5 335)**	**Improved [b)] (n = 3 919)**
**Age in years**	54.5 (12.2)	54.6 (11.9)
**BMI (kg/m²)**	28.1 (4.7)	27.7 (4.5)
**eCRF (ml/kg/min)**	33.6 (7.0)	34.4 (6.9)
**Bodily pain [c)]**	4.17 (0.39)	4.38 (0.56)
**Sex**		
Women	3 424 (60.1)	2 277 (39.9)
Men	1 911 (53.8)	1 642 (46.2)
**eCRF quintiles**		
1st	1 064 (60.1)	708 (39.9)
2nd	880 (59.1)	608 (40.9)
3rd	787 (56.5)	607 (43.5)
4th	660 (53.7)	570 (46.3)
5th	586 (54.9)	481 (45.1)
**Organ disease [d)]**		
No	2 070 (53.3)	1 816 (46.7)
One	1 719 (57.9)	1 250 (42.1)
Two or more	1 171 (65.5)	618 (34.5)
**HADS-D**		
HADS-D < 8	3 759 (56.9)	2 848 (43.1)
HADS-D ≥ 8	597 (59.5)	407 (40.5)
**HADS-A**		
HADS-A < 8	3 347 (56.3)	2 599 (43.7)
HADS-A ≥ 8	995 (60.6)	646 (39.4)
**Smoking status**		
Never	1 839 (57.9)	1 338 (42.1)
Former	1 981 (57.3)	1 478 (42.7)
Current	1 429 (58.3)	1 021 (41.7)
**In employment [e)]**		
Yes	4 250 (57.3)	3 163 (42.7)
No	1 069 (59.1)	740 (40.9)

Data are presented as mean and standard deviation (SD) for continuous variables and as numbers (row percentages) for categorical variables. Abbreviations: BMI (kg/m²) = body mass index, calculated as weight in kilograms divided by height in meters squared; eCRF = estimated cardiorespiratory fitness; eCRF quintiles = quintiles of eCRF, where the first quintile represents the 20% of participants with the lowest eCRF within each age- and sex-specific group; HADS-D = Hospital Anxiety and Depression Scale – Depression subscale; HADS-A = Hospital Anxiety and Depression Scale – Anxiety subscale. Footnotes: [a)] no improvement or worsening in pain; [b)] decrease in pain severity by ≥1 point on the 6-point Bodily Pain Scale (SF-8). [c)]Bodily Pain Scale 1 = no pain; 2 = very mild pain; 3 = mild pain; 4 = moderate pain; 5 = severe pain; 6 = very severe pain. [d)] Based on self-report of the following: myocardial infarction, angina pectoris, other heart disease, stroke/brain haemorrhage, kidney disease, diabetes, cancer, epilepsy, chronic bronchitis, asthma, ankylosing spondylitis, rheumatoid arthritis, or osteoarthritis. [e)] Among participants aged 20–64 (working population).

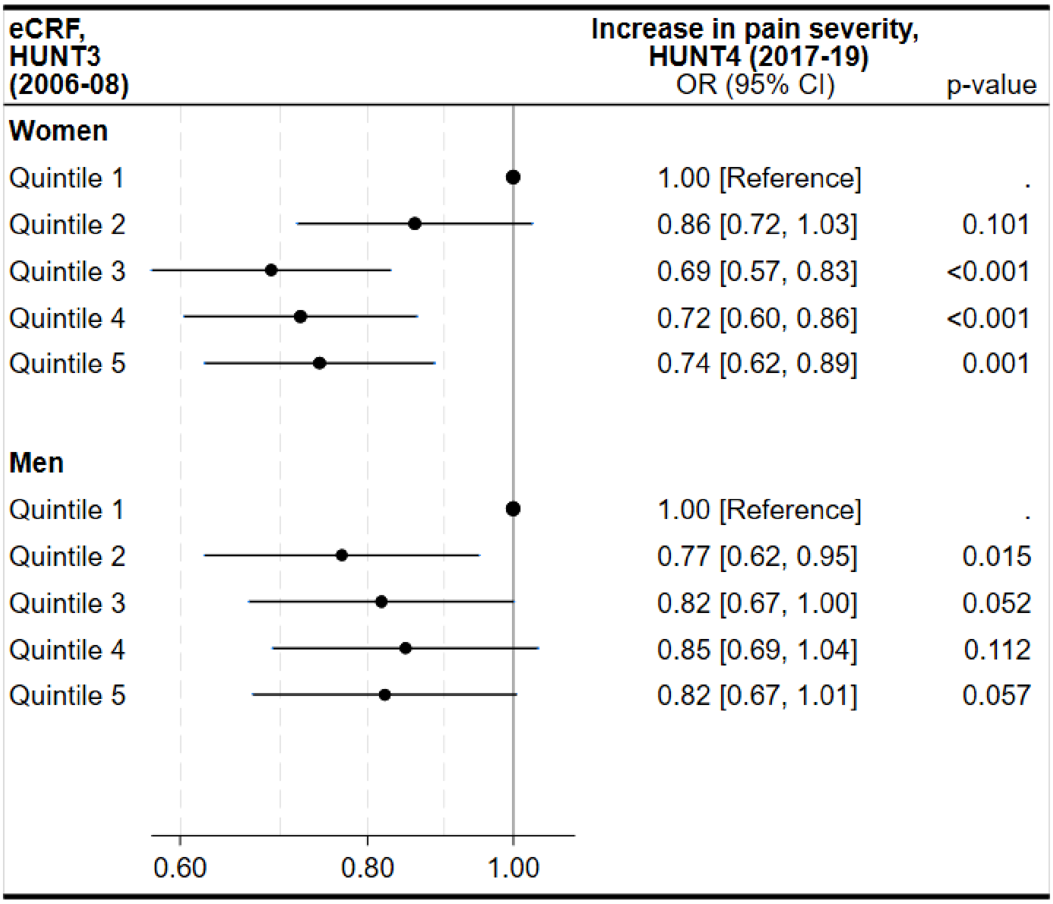

Supplementary Figure 1*: Longitudinal associations between quintiles of estimated cardiorespiratory fitness (eCRF) and a ≥2-point increase in pain severity, presented for women and men. Odds ratios (ORs) and 95% confidence intervals (CI) are shown for each eCRF quintile, with Quintile 1 as the reference. OR are adjusted for age, organ diseases (myocardial infarction, angina pectoris, other heart diseases, stroke/brain hemorrhage, kidney disease, diabetes, cancer, epilepsy, chronic bronchitis, asthma, ankylosing spondylitis, rheumatoid arthritis, or osteoarthritis), anxiety and depressive symptoms (HADS-D, HADS-A), smoking status, and work status.*

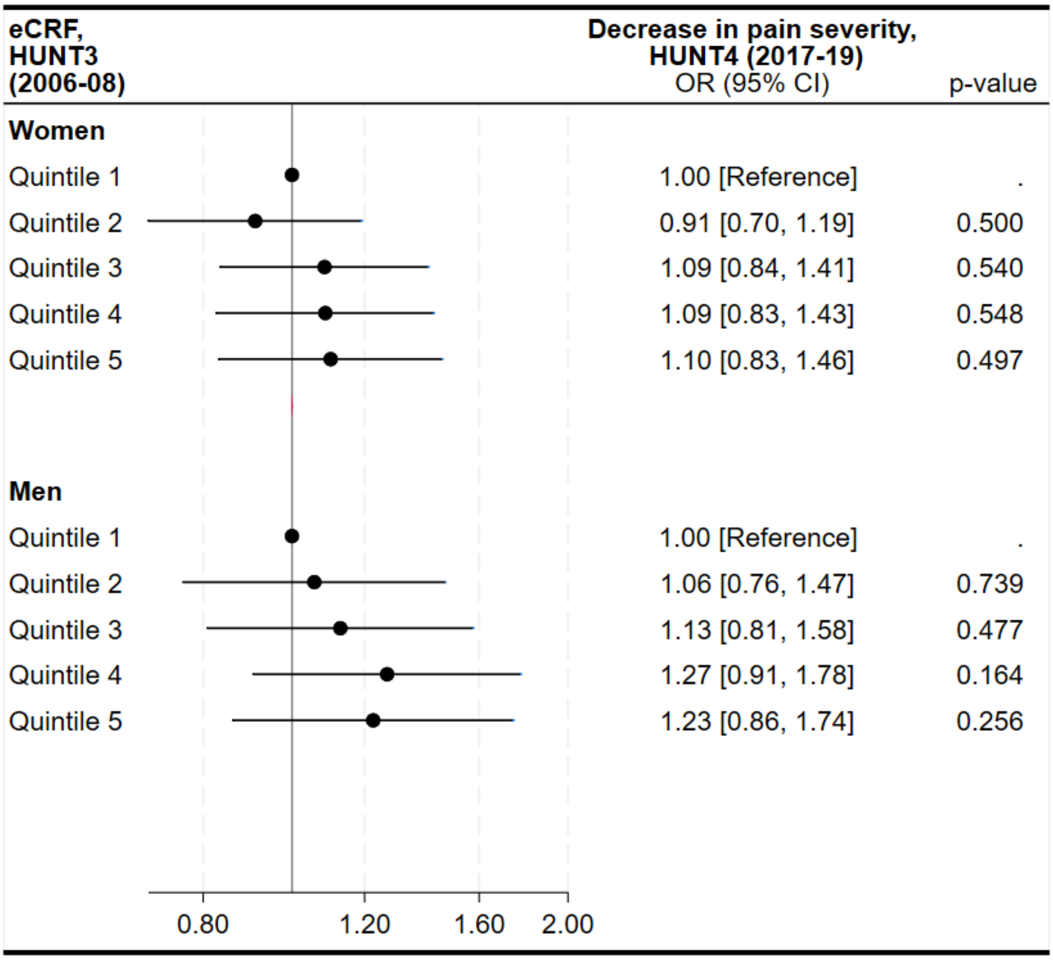

Supplementary Figure 2: *Longitudinal associations between quintiles of estimated cardiorespiratory fitness (eCRF) and a ≥2-point decrease in pain severity, presented for women and men. Odds ratios (ORs) and 95% confidence intervals (CI) are shown for each eCRF quintile, with Quintile 1 as the reference. OR are adjusted for age, organ diseases (myocardial infarction, angina pectoris, other heart diseases, stroke/brain hemorrhage, kidney disease, diabetes, cancer, epilepsy, chronic bronchitis, asthma, ankylosing spondylitis, rheumatoid arthritis, or osteoarthritis), anxiety and depressive symptoms (HADS-D, HADS-A), smoking status, and work status.*

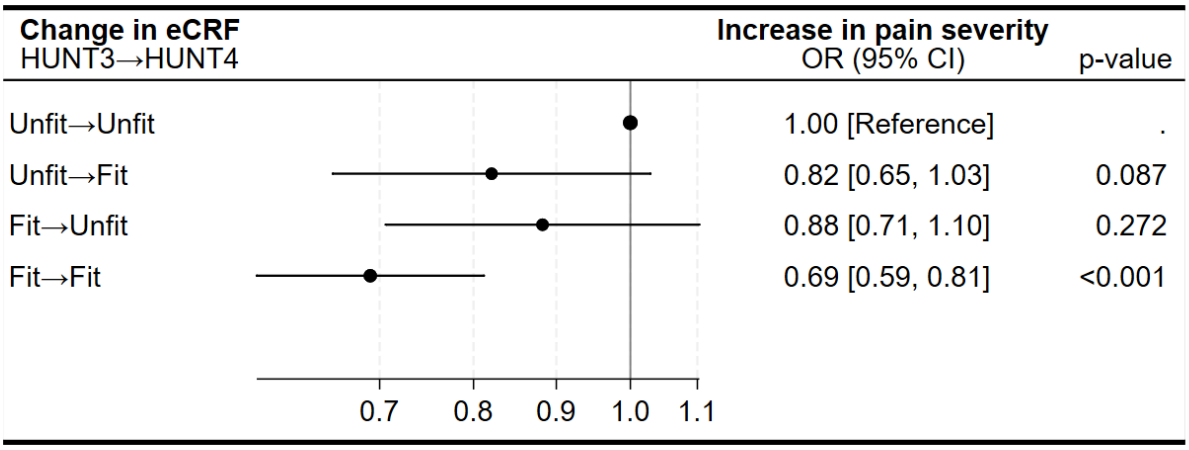

Supplementary Figure 3: *Longitudinal associations between changes in estimated cardiorespiratory fitness (eCRF) and a ≥2 change (increase) in pain severity among participants with no or mild pain at baseline. eCRF categories were defined as unfit (≤20% of participants) and fit (>20% of participants). Odds ratios (ORs) and 95% confidence intervals (CI) are shown for each eCRF change category, with the "unfit→unfit" group as the reference. Odds ratios (ORs) are adjusted for age, organ diseases (myocardial infarction, angina pectoris, other heart diseases, stroke/brain hemorrhage, kidney disease, diabetes, cancer, epilepsy, chronic bronchitis, asthma, ankylosing spondylitis, rheumatoid arthritis, or osteoarthritis), anxiety and depressive symptoms (HADS-D, HADS-A), smoking status, and work status.*

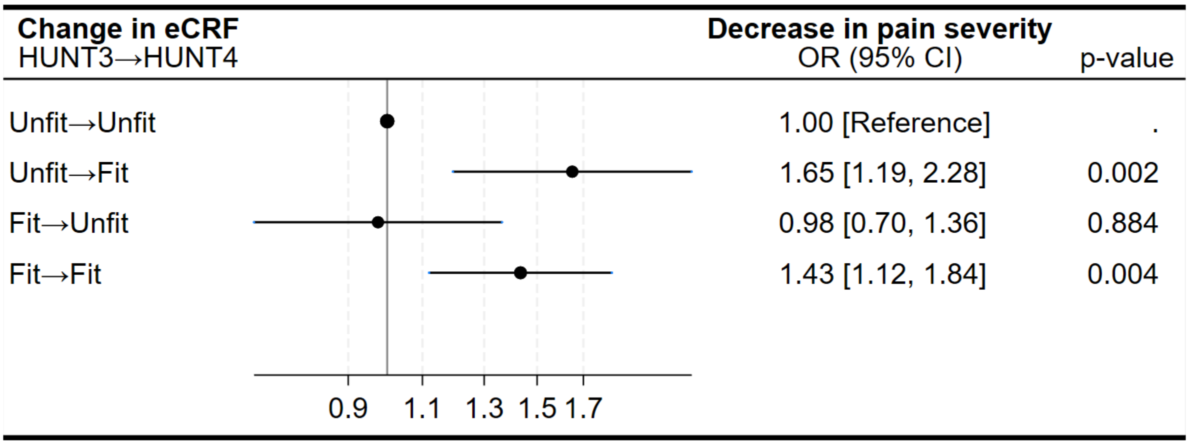

Supplementary Figure 4*: Longitudinal associations between changes in estimated cardiorespiratory fitness (eCRF) and a ≥2 changes (decrease) in pain severity among participants with moderate to severe chronic pain at baseline. eCRF categories were defined as unfit (≤20% of participants) and fit (>20% of participants). Odds ratios (ORs) and 95% confidence intervals (CI) are shown for each eCRF change category, with the "unfit→unfit" group as the reference. Odds ratios (ORs) are adjusted for age, organ diseases (myocardial infarction, angina pectoris, other heart diseases, stroke/brain hemorrhage, kidney disease, diabetes, cancer, epilepsy, chronic bronchitis, asthma, ankylosing spondylitis, rheumatoid arthritis, or osteoarthritis), anxiety and depressive symptoms (HADS-D, HADS-A), smoking status, and work status.*

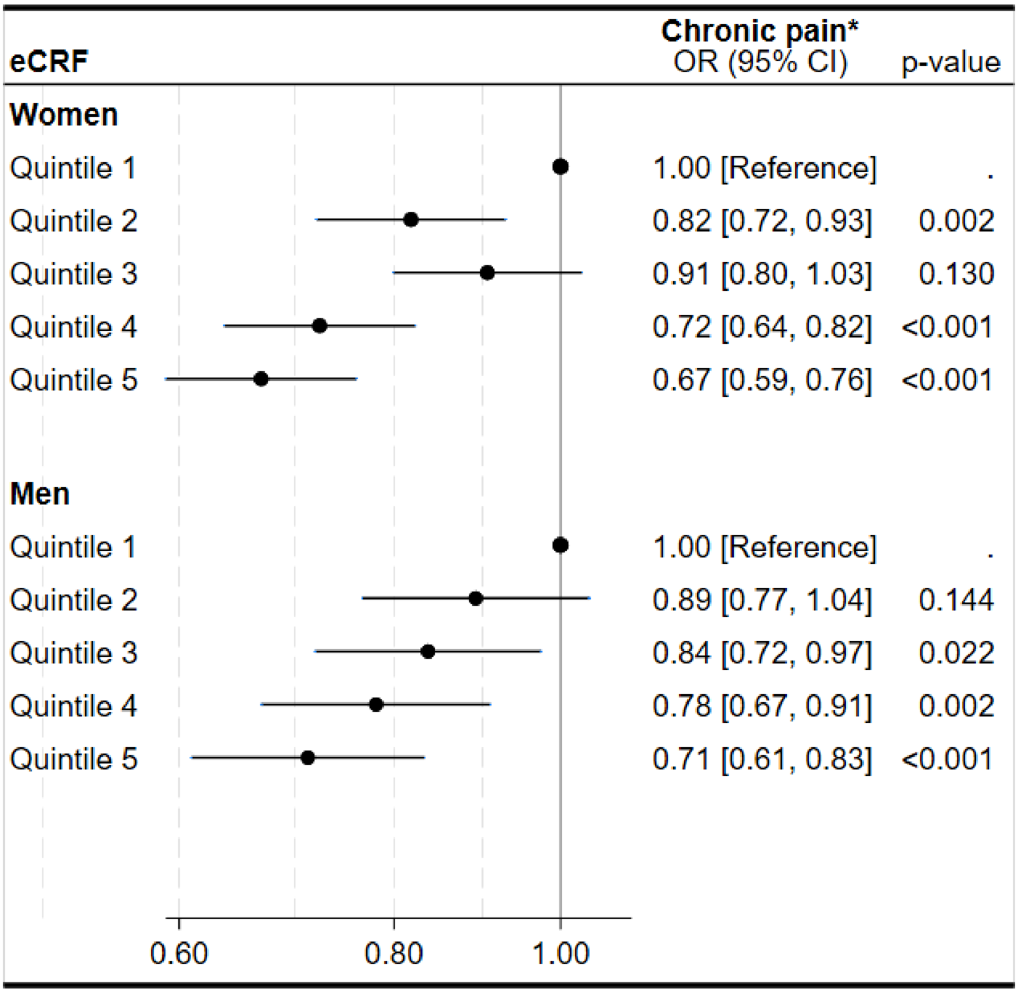

Supplementary Figure 5*: Cross-sectional associations between quintiles of estimated cardiorespiratory fitness (eCRF) and pain lasting more than 6 months, presented for women (n = 11 714) and men (n = 8 138). Odds ratios (ORs) and 95% confidence intervals (CIs) are shown for each eCRF quintile, with Quintile 1 as the reference. ORs are adjusted for age, organ diseases (myocardial infarction, angina pectoris, other heart diseases, stroke/brain hemorrhage, kidney disease, diabetes, cancer, epilepsy, chronic bronchitis, asthma, ankylosing spondylitis, rheumatoid arthritis, or osteoarthritis), anxiety and depressive symptoms (HADS-D, HADS-A), smoking status, and work status. * Duration-only criterion (6months), irrespective of pain severity.*

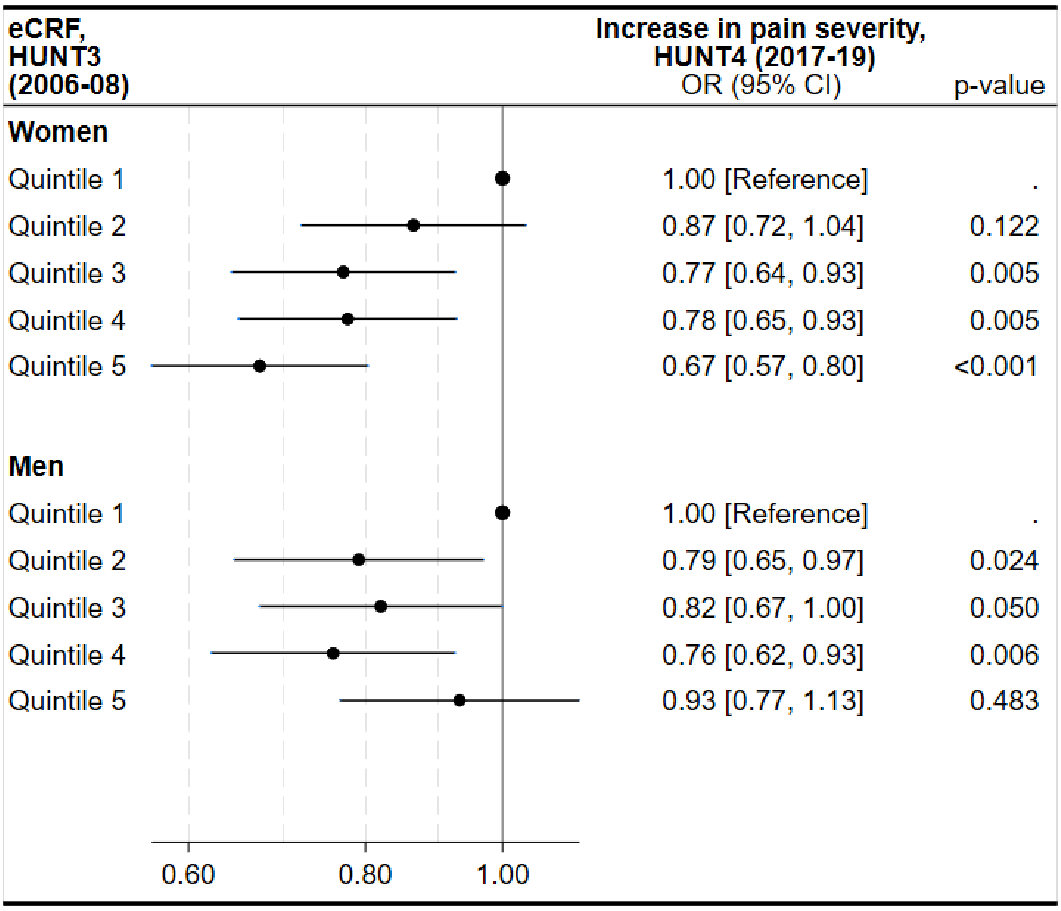

Supplementary Figure 6*: Longitudinal associations between quintiles of estimated cardiorespiratory fitness (eCRF) and change (increase) in pain severity among participants with no pain or acute mild pain at baseline (i.e. excluding those with chronic mild pain), presented for women (n = 5 406) and men (n = 4 268). Odds ratios (ORs) and 95% confidence intervals (CIs) are shown for each eCRF quintile, with Quintile 1 as the reference. ORs are adjusted for age, organ diseases (myocardial infarction, angina pectoris, other heart diseases, stroke/brain hemorrhage, kidney disease, diabetes, cancer, epilepsy, chronic bronchitis, asthma, ankylosing spondylitis, rheumatoid arthritis, or osteoarthritis), anxiety and depressive symptoms (HADS-D, HADS-A), smoking status, and work status.*
